# Laparoscopic Treatment of a Rare Right Diaphragmatic Rupture with Small Bowel Herniation after Blunt Thoracic Trauma

**DOI:** 10.1155/2010/109062

**Published:** 2010-06-28

**Authors:** H. Hoffmann, D. Oertli, O. Heizmann

**Affiliations:** Department of Surgery, University Hospital Basel, 4031 Basel, Switzerland

## Abstract

Blunt traumatic diaphragmatic rupture (BTDR) is a life-threatening condition with an incidence from 0,8%–1,6% in blunt trauma, mostly located on the left side. The main prognostic factors are severe side injuries and the delay of diagnosis. We present a rare case of a 68-year-old female, with an isolated right diaphragm rupture. The diagnosis was done with a delay of 4 days by thoracic radiographs, which showed a herniation of small bowel into the right thoracic cavity. A reposition of the small bowel and a closure of the diaphragmatic defect by running suture were carried out laparoscopicly. Although large prospective studies concerning the outcome of laparoscopic approach to right BTDR are still missing, we could show, that laparoscopy can be performed safely in right traumatic diaphragm rupture.

## 1. Introduction

Blunt traumatic diaphragmatic rupture (BTDR) is a life-threatening condition with an incidence of 0.8%–1.6% in blunt trauma [1–3]. The closure of the diaphragm rupture must be performed immediately. The diagnosis often happens to be late due to the absence of typical symptoms or other major injuries dominating the clinical aspect [4]. An isolated BTDR is rare and thus might be followed by a period of weeks or months not revealing any symptoms [2, 5]. Most BTDR are located on the left side in the musculotendinous intersection [1, 3, 4]. Right BTDR are rarely described and less frequent [6]. Herniation of colon, small bowel, or liver may occur and result in ileus, necrosis, and perforation [4, 7].

## 2. Narrative

**Figure 1:**
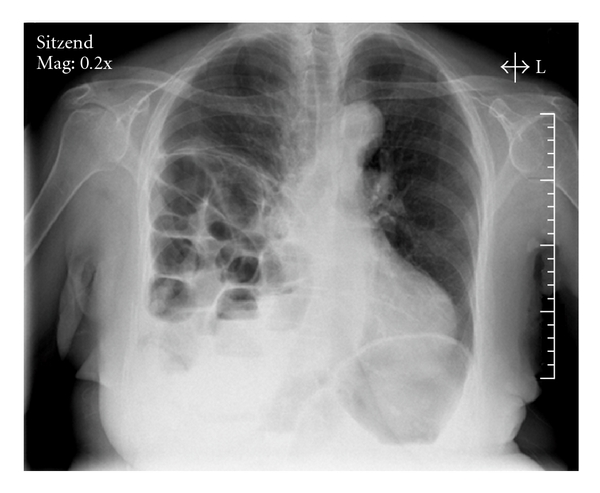
Chest radiograph shows bowel herniation into right hemithorax.

We present a rare case of a 68-year-old female hospitalized in the neurological department due to Parkinson disease. She fell onto a chair hitting her right hemithorax. Initially, subjective symptoms have been missing. The examination showed a slightly reduced breath without any signs of pneumothorax or dyspnea, a decent pressure pain and a bruise. 4 days after trauma, she developed a progressive pulmonal decompensation with desaturation. Additionally, there have been signs of ileus. The chest radiograph displayed a herniation of bowel into the right hemithorax with consecutive ileus signs (Figure 1). 

**Figure 2:**
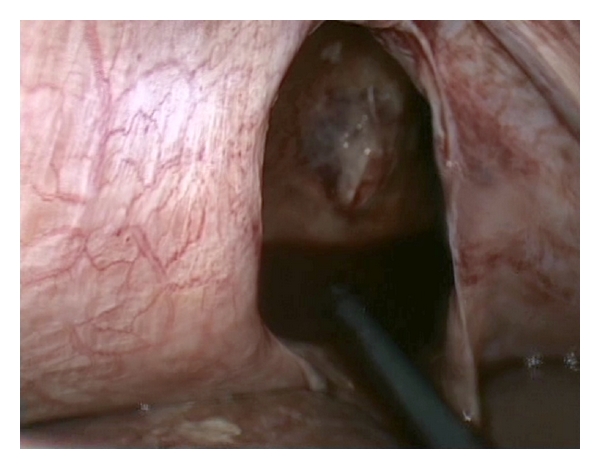
Transdiaphragmatic view into the right thoracic cavity.

We performed a laparoscopic approach and found a 4 × 5 cm rupture of the right diaphragm with herniation of 1 meter small bowel. The bowel appeared vital after reposition. The transdiaphragmatic thoracoscopy displayed a collapsed lung and a dislocated rib fracture (Figure 2). After irrigation of the thoracic cavity we made a direct laparoscopic strainless running suture with nonabsorbable tie (0/0 Ethibond). A drain was positioned in the right hemithorax. Afterwards the patient showed an uneventful course.

## 3. Discussion

The delay of the diagnosis was 4 days indicating the variety of the clinical appearance of the BTDR. The missing typical symptoms can mask the severity of the injury and lead to increasing morbidity. Although large prospective long-term studies regarding outcome after laparoscopic approach are still missing [1], laparoscopy was the method of choice in our case and safely performed, particularly when severe side injuries are absent. We could show, that laparoscopy has advantages in isolated diaphragmatic ruptures compared to traditional laparotomy, which is still preferred by some authors [8, 9]. Laparoscopy mostly allows easy reposition of herniated organs, sufficient inspection of the thoracic, and abdominal cavity and immediate laparotomy if necessary.
